# *Limosilactobacillus reuteri* B1/1 modulated the intestinal immune response in preventing *Salmonella* Enteritidis PT4 infection in a chicken ileal explant model

**DOI:** 10.1007/s11259-024-10609-4

**Published:** 2024-11-23

**Authors:** Viera Karaffová, Zuzana Kiššová, Csilla Tóthová, Patrik Tráj, Máté Mackei, Gábor Mátis

**Affiliations:** 1 Department of Morphological Disciplines, University of Veterinary Medicine and Pharmacy in Košice, Komenského 73, Košice, 040 01 Slovakia; 2 Clinic of Ruminants, University of Veterinary Medicine and Pharmacy in Košice, Komenského 73, Košice, 041 81 Slovakia; 3 Division of Biochemistry, Department of Physiology and Biochemistry, University of Veterinary Medicine, István utca 2, Budapest, H-1078 Hungary

**Keywords:** Ileal explant, *salmonella*, *Limosilactobacillus*, Immune response, *Ex vivo*

## Abstract

In this study, we observed the effect of the newly isolated probiotic strain *Limosilactobacillus reuteri* B1/1 on the relative gene expression of selected cytokines (interleukin-15, transforming growth factor-β4), tight junction proteins (E-cadherin, occludin), biomarker active intestinal stem cells - LGR5 (leucine-rich repeat containing G protein-coupled receptor), markers of mucosal intestinal immunity (mucin-2, immunoglobulin A), as well as the creation of a new biomarker of inflammation in the intestine - calprotectin on an ex vivo model of chicken ileal explant in the prevention of *Salmonella* Enteritidis PT4 infection. The ability of *L. reuteri* B1/1 to effectively modulate the mucosal immune response under pretreatment conditions in S. Enteritidis PT4 infection in a chicken ileal explant model was confirmed. In addition, our obtained results point to the fact that the new chicken ileum explant model could be a suitable model to investigate or test the influence of natural substances such as probiotic bacteria in the interaction with the intestine as well as pathogenic microorganisms. In addition, the results of our study may contribute to a deeper understanding of the action of newly isolated probiotic bacteria at the intestinal level using ex vivo models such as chicken ileum explant, which are able to mimic in vivo conditions sufficiently.

## Introduction

Intensive production systems realize the majority of poultry meat production, while extensive poultry rearing systems (free-range or organic) represent only a small part of poultry production in the EU (approximately 5%) (Dal Bosco et al. 2021). The important fact remains that broiler chickens on intensive production farms live in a high-density environment and are thus exposed to an increased risk of gastrointestinal (GI) infections due to gut microbiome imbalances. Moreover, global production of this product is driven by the demand for poultry meat, which is cheaper than red meat. The aforementioned adverse conditions can weaken the immune system, leading to reduced productivity and, ultimately, increased mortality (Korver 2023; Diaz Carrasco et al. 2019).

Currently, according to the results of zoonoses surveillance reported annually by EFSA and ECDC, foodborne infections in the EU are mainly caused by *Campylobacter* and *Salmonella* strains, with poultry products (poultry meat and eggs) being the primary source of human infection (EFSA 2023). In addition, the first choice of treatment is the use of antimicrobial therapy, which, due to incorrect and excessive use, has led to the development of antimicrobial resistance in animal production, which poses a direct threat to consumers as well as to human society in general (Castro-Vargas et al. 2020).

Accordingly, scientific research on a global scale focuses on the search for possible alternative solutions, also in the form of the use of natural substances such as probiotics, which are becoming an increasingly current trend.

Generally, the application of probiotic bacteria has a beneficial effect on animals as well as humans health through many mechanisms such as modulation of digestive processes, improvement of nutrient utilization, production parameters, regulation of immune reactions, antimicrobial activity (Dempsey and Corr 2022; Un-Nisa et al. 2022; Latif et al. 2023). Especially in poultry farms, when lactobacilli were used in feed, a reduction in nutrient requirements was noted through the mechanism of increased utilization of nitrogen and phosphorus, which facilitates digestion. At the same time, there was an increase in the innate and acquired immunity of poultry. Several recent studies indicate that lactobacillus probiotic strains were able to influence the innate immunity of poultry by modulating the proliferation of heterophils, macrophages as well as B-lymphocytes, thereby significantly contributing to protection against pathogenic microorganisms (Abd El-Hack et al. 2020; Jeni et al. 2021; Racines et al. 2023).

It is widely known that *L. reuteri* is a resident probiotic strain found in the intestines of animals, with a strong ability of intestinal adhesion and colonization. *L. reuteri* B1/1 a novel probiotic isolate from the intestine of healthy pheasants, has been sufficiently characterized, resulting in the current process of depositing it in a collection of microorganisms (Kiššová et al. 2024). However, there is still room to explore the possibilities of its use in animal nutrition. In addition, according to our recent researches, it showed a promising immunomodulating effect (Karaffová et al. 2020; Karaffová et al. 2023; Kiššová et al. 2024).

From this point of view, it should be emphasized that the gastrointestinal tract, especially the small intestine, plays a vital role in defense including nutrient absorption, digestion, immunity, as well as product performance. Intestinal homeostasis is maintained by stem cells located in the crypts, which produce cells that have the ability to differentiate into cell types, such as goblet cells, Paneth cells, tuft cells, and enteroendocrine cells as needed (Kang and See 2024). Cells produce various factors, markers and cytokines including e-cadherin, occludin, LGR5 receptor protein, IgA, mucin-2 (MUC-2) that participate in maintaining the intestinal barrier and protect against invasion by various pathogens (including *salmonella*) and adverse environmental factors (Lee and Kim 2018).

Calprotectin is a protein biomarker found in white blood cells, the gut, and released in the stool during intestinal inflammation. It has strong antibacterial and antifungal properties (Pathirana et al. 2018). To the best of our knowledge, only a few studies (De Meyer et al. 2019; Dal Pont et al. 2021) focused on monitoring the production of calprotectin in the intestine or the poultry excreta. Not to mention the influence of probiotic bacteria on the production of calprotectin, which can be used as a diagnostic tool for diseases of the digestive tract of poultry. Therefore, in this sense, it may be interesting to observe the effect of applied probiotic bacteria such as *L. reuteri* B1/1 on the production of calprotectin during inflammation in the poultry intestine.

The European Union in terms of animal welfare regulations (the ARRIVE guidelines) requires, among other things, a reduction in the number of animals used in an experiment (Kilkenny et al. 2010). In accordance with the above, in this study we focused on the use of an ex vivo ileal explant model from broiler chicken, which was methodically established by a team led by Assoc. Prof. Mátis from the University of Veterinary Medicine in Budapest (Mátis et al. 2024b). This type of model can represent a suitable variant of the experimental model, as it allows us, among other things, to reduce the amount of animals used in experiments, which is in accordance with the regulations.

Therefore, we decided to observe the effect of *L. reuteri* B1/1 on relative gene expression of selected cytokines (IL-15, TGF-β4), tight junction (TJ) proteins (E-cadherin, occludin), biomarker of active intestinal stem cells - LGR5 (leucine-rich repeat containing G protein-coupled receptor), markers of mucosal intestinal immunity (MUC-2, IgA) as well as the production of the new biomarker of inflammation in the intestine - calprotectin on an ileal chicken explant model in prevention of *Salmonella* Enteritidis PT4 infection.

## Materials and methods

### Explant sample isolation

A one 3-week-old male Ross-308 broiler sacrificed in accordance with the animal welfare legislation of the European Union and the guidelines required by the Local Animal Welfare Committee of the University of Veterinary Medicine in Budapest. Approval for the experiment was obtained from the Government Office of Zala County, Plant Protection, Food Chain Safety, and Soil Conservation Directorate, Zalaegerszeg, Hungary (May 11, 2020; GK-419/2020). All explants were isolated from one animal in order to maintain the homogeneity of the samples. The small intestines were collected after aseptically opening the coelomic cavity in the dorsal position.

The methodology for the isolation of gut explants from the ileum followed the previously established protocol developed by Mátis et al. (2024b). Approximately a 13 cm long ileal segment, positioned distally from the Meckel’s diverticulum was removed from a Ross-308 broiler after decapitation. In summary, ileal explants were excised using 1.5 mm diameter biopsy punches with plungers (WPI, Sarasota, USA) after the removal mesenteric fat, repeated internal and external washing with sterile phosphate-buffered saline (PBS) supplemented with 1% penicillin-streptomycin solution (Gibco, Waltham, MA USA) and lengthwise opening of the segment. Finally, 96-well culture plates (Greiner Bio One Hungary Kft. in Mosonmagyaróvár, Hungary) prefilled with Dulbecco’s modified Eagle medium/F-12 Ham nutrient medium (Sigma-Aldrich, Missouri, USA) were seeded with ileal explants.

#### Bacterial strains

##### ***Limosilactobacillus reuteri *B1/1**

The probiotic strain *L. reuteri* B1/1 (LR) (GenBank PP911451.1) was grown in de Man Rogosa Sharpe (MRS) broth (Merck, Darmstadt, USA) at 37 °C overnight. Bacteria were then centrifuged at 500 x g for 10 min, the pellets were washed three times in PBS and resuspended in DMEM/F12 medium without antibiotics (Sigma-Aldrich, St. Louis, Missouri, USA).

***Salmonella*** **Enteritidis PT4**

*Salmonella enterica* serovar Enteritidis (*S*. Enteritidis; SE) phage type PT4 was provided from doc. RNDr. Ivan Rychlík, Ph.D (Veterinary Research Intitute, Brno, Czech republic). *Salmonella* was cultured in Luria–Bertani broth (LB; Sigma-Aldrich, St. Louis, Missouri, USA) with continuous shaking (160 rpm) overnight at 37 °C. Overnight cultures inoculated into fresh LB medium were then incubated for 4 h at 37 °C with permanent shaking.

The bacterial concentration was quantified by measuring the optical density (OD) at 600 nm in a Synergy HTX Multi-Mode Reader spectrophotometer (Agilent, Santa Clara, CA, USA). Additionally, bacterial concentration was confirmed by serial dilution and determination of colony-forming units (CFU) on Mueller-Hinton agar plates (Sigma-Aldrich, St. Louis, Missouri, USA). Bacteria were diluted in serum- and antibiotic-free culture medium to the desired concentration before addition to the ileal explants.

### Design of experiments

A total of 4 groups were used in the experiment and 12 replicates per treatment. The control group of ileal explants was incubated in DMEM/F12 medium without supplementation (Sigma-Aldrich, St. Louis, Missouri, USA). In the LR experimental group, explants were incubated in DMEM/F12 medium containing *L. reuteri* 1 × 10^8^ CFU/ml for 2 h (37 °C, 5% CO_2_). In the SE PT4 infected group, explants were treated with culture medium containing *Salmonella* Enteritidis PT4 at a concentration of 1 × 10^7^ CFU/ml, incubated for 2 h (37 °C, 5% CO_2_). In the pre-treatment experimental group, explants were first incubated with *L. reuteri* at a concentration of 1 × 10^8^ CFU/ml for 2 h (37 °C, 5% CO_2_), followed by exposure to SE for 2 h (1 × 10^7^ CFU/ml) (37 °C, 5% CO 2). After incubation, explants from each group were washed with 100 µg/ml gentamicin solution (Sigma-Aldrich, Missouri, USA) diluted in DMEM/F12.

### Homogenization of explant samples and isolation of total RNA

Ileal explant samples were placed in RNA Later solution (Qiagen, Hilden, Germany) and stored at − 70 °C until RNA purification and transcription were carried out as described in Karaffová et al. (2017).

### Gene expression analysis - real Time-qPCR

The relative gene expression of cytokine (IL-15, TGF-B4), E-cadherin, OCCL (occludin), LGR5 and IgA, MUC-2 as well as reference gene GAPDH (glyceraldehyde-3-phosphate dehydrogenase) was determined using Real Time - qPCR.

Amplification and detection of transcripts were performed using the Power SYBR™ Green PCR Master Mix (Thermo Scientific, USA) and specific primers (Table 1) on a LightCycler 480 II (Roche, Switzerland) according to a predefined temperature program: initial denaturation at 95 °C for 5 min; followed by 39 cycles of amplification: denaturation 94 °C/30 s, hybridization 60 °C/30 s, extension at 72 °C/30 s and final extension 72 °C/15 min. A melting curve from 50 °C to 95 °C with readings at every 0.5 °C was noted for each individual RT-qPCR plate. Analysis was performed after every run to ensure a single amplified product for each reaction. All reactions for qPCR were performed in duplicate. All primer sets allowed cDNA amplification efficiencies between 94% and 100%. It was confirmed that the efficiency of amplification for each target gene (including GAPDH) was essentially 100% in the exponential phase of the reaction, where the quantification cycle (Cq) was calculated. The Cq values of the studied genes were normalised to an average Cq value of the reference gene (ΔCq), and the relative expression of each gene was calculated mathematically as 2–^ΔΔCq^.

**Table 1:** List of primers used for the gene mRNA quantification in ileal explants

Primer	Sequence 5’–3’	References
IL-15 Fw	TGGAGCTGATCAAGACATCTG	Kolesárová et al. 2011
IL-15 Rev	CATTACAGGTTCCTGGCATTC	
TGFB4 Fw	AGGATCTGCAGTGGAAGTGGA	Swaggerty et al. 2004
TGFB4 Rev	CCCCGGGTTGTGTGTTGG TGGT	
E-cadherin Fw	TGAAGACAGCCAAGGGCCTG	In this study
E-cadherin Rev	CTGGCGGTGGAGAGTGTGAT	
OCCL Fw	TGCTTTTGCCCAAGCAGGAA	Ghiselli et al. 2021
OCCL Rev	TGTGGGAGAGGCACCAGTTG	
LGR5 Fw	TGGGCTCCACAGCCTAGAGA	
LGR5 Rev	CCTACAAACGCACGCTCAGG	
IgA Fw	GTCACCGTCACCTGGACTACA	Lammers et al. 2010
IgA Rev	ACCGATGGTCTCCTTCACATC	
MUC-2 Fw	GCTGATTGTCACTCACGCCTT	Smirnov et al. 2006
MUC-2 Rev	ATCTGCCTGAATCACAGGTGC	
GAPDH Fw	CCTGCATCTGCCCATTT	De Boever et al. 2008
GAPDH Rev	GGCACGCCATCACTATC	

### Sequence data Collection

Chicken E-cadherin gene sequences were retrieved from 3 public nucleic acid databases including GenBank (http://www.ncbi.nlm.nih.gov/), Silva comprehensive ribosomal RNA database (Silva, http://www.arb-silva.de/), and Ribosomal Database Project (RDP, http://rdp.cme.msu.edu/) in January 2024, using the following search terms: chick, chicks, poultry, broiler, and layer. Possible chimeric sequences were found using via Chimera Slayer and UCHIME in the Mothur package (Schloss et al. 2009; Edgar et al. 2011) and were removed. The database record information associated with each of the sequences was evaluated, and the sequences not of poultry gut origin were removed manually.

### Laboratory analysis - ELISA

Chicken calprotectin was analyzed using MBS7606348 chicken-specific double antibody sandwich ELISA test (MyBioSource, San Diego, CA, USA) in microplates. Calibration was performed using a set of standards from the kit at a range of the following concentrations: 40, 20, 10, 5, 2.5, 1.25 and 0.625 ng/ml. The sensitivity of the kit was 0.375 ng/ml. The samples were diluted 1:2 with Sample Dilution Buffer provided in the kit. The absorbance was read on Opsys MR automatic microplate reader (The Dynex Technologies, USA) at a wavelength of 450 nm. The Revelation QuickLink version 4.25 computer software was used for the calculation of results (The Dynex Technologies, USA).

### Statistical analysis

The Shapiro–Wilk test was used to analyse data normality of gene expression. Since the data showed normality, they were tested by one-way ANOVA followed by post hoc multi-comparison Tukey’s test using Graph Pad Prism version 8.00 (GraphPad Software Inc., California, USA) to determine differences between each group. Differences between the mean values for each group were considered statistically significant at *p* < 0.05^*ab*^; *p* < 0.01^*ac*^; *p* < 0.001^*ad*^; *p* < 0.0001^*ae*^. Values in figures are given as means with standard deviations (± SD).

Statistical analysis of calprotectin production was determined by descriptive statistics using the GraphPad Prism V5.02 computer program (GraphPad Software Inc., California, USA). Kolmogorov-Smirnov test for normality was applied to evaluate the distribution of the data. The data were not normally distributed. Therefore, the significance of differences between the groups was analyzed by Kruskal-Wallis Test with Dunn’s Multiple Comparison Test. Differences were considered statistically significant at *p* < 0.001^*ad*^; *p* < 0.0001^*ae*^. Values in figure are given as median with standard deviations (± SD).

### Result

**Fig. 1:**
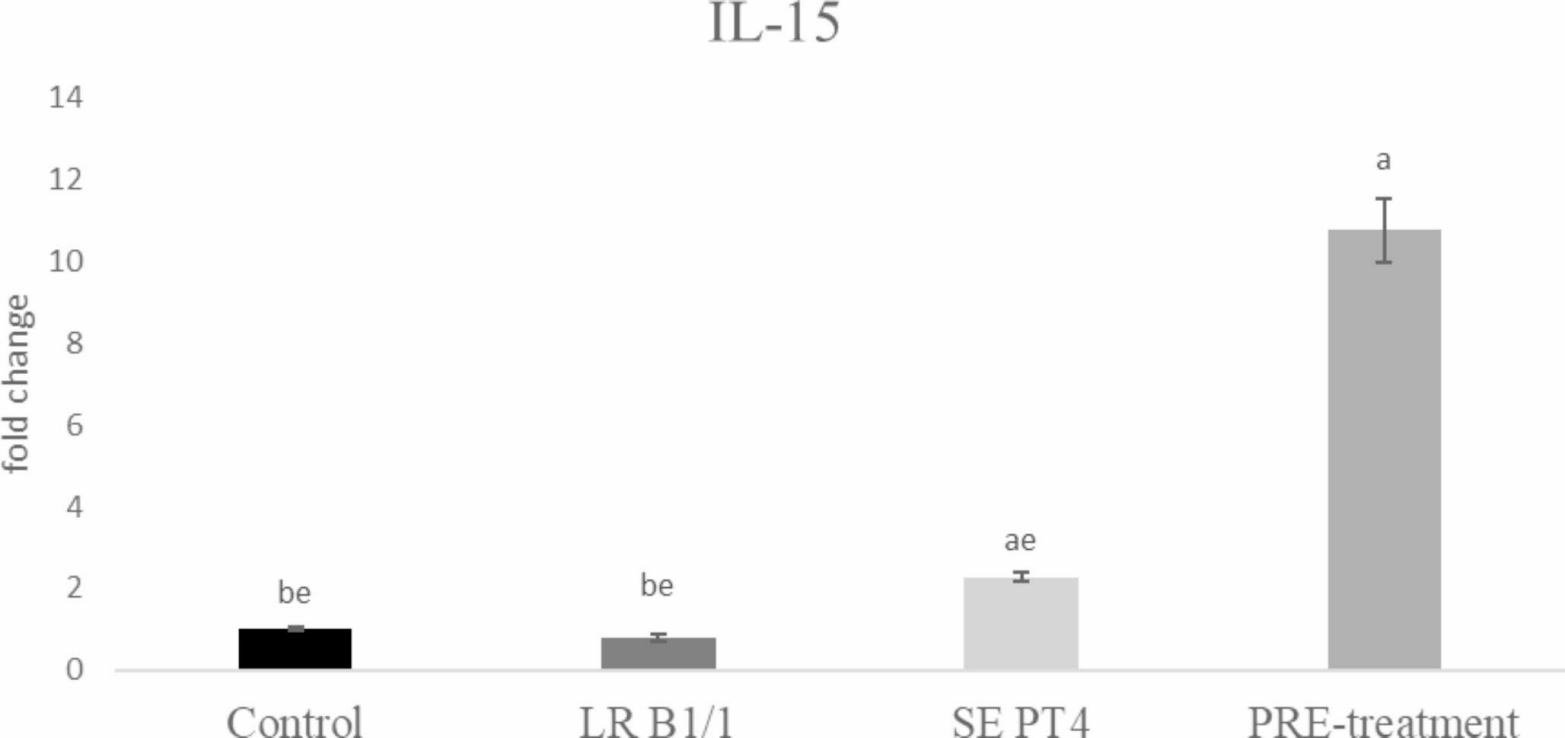
Relative gene expression for IL-15 in ileal explant. ^a−b^ different letters indicate significant differences among groups and time points at *p* < 0.05;^a−e^ different letters indicate significant differences among groups and time points at *p* < 0.0001;

**Fig. 2:**
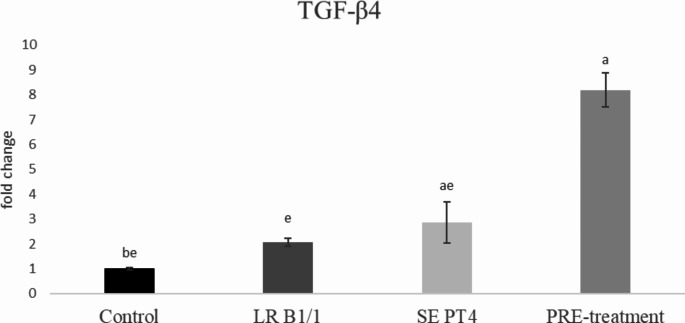
Relative gene expression for TGF-β4 in ileal explant. ^a−b^ different letters indicate significant differences among groups and time points at *p* < 0.05;^a−e^ different letters indicate significant differences among groups and time points at *p* < 0.0001

**Fig. 3:**
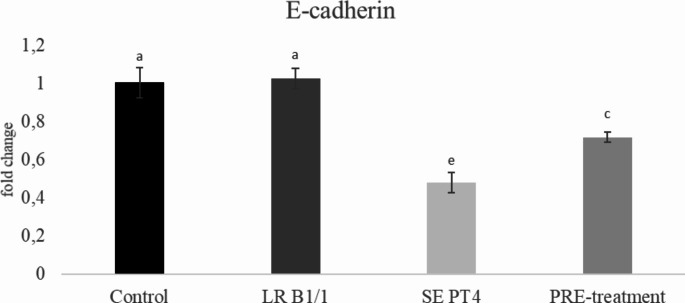
Relative gene expression for E-cadherin in the ileal explant. ^a−c^ different letters indicate significant differences among groups and time points at *p* < 0.01; ^a−e^ different letters indicate significant differences among groups and time points at *p* < 0.0001

**Fig. 4:**
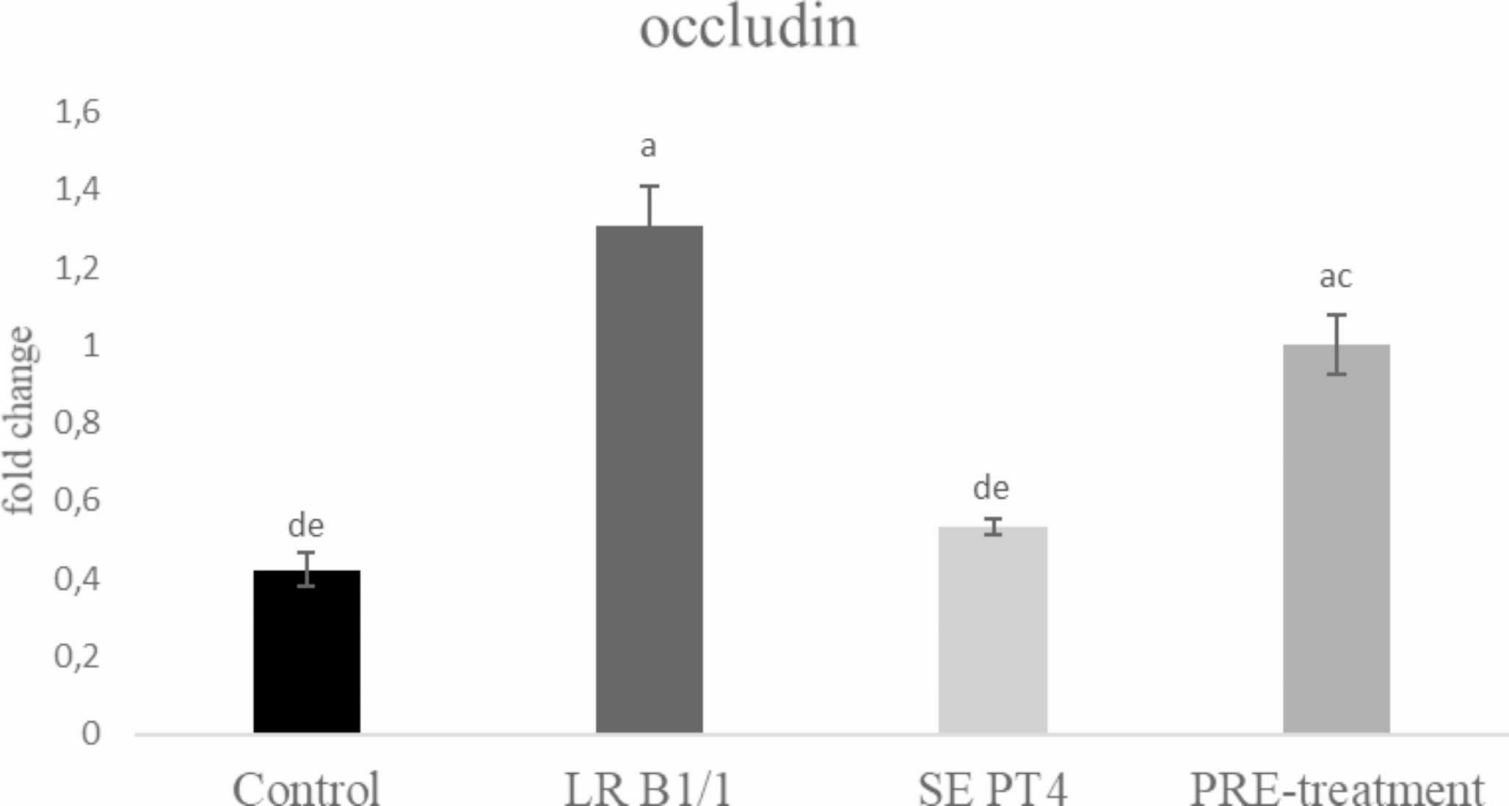
Relative gene expression for occludin in the ileal explant. ^a−c^ different letters indicate significant differences among all groups and time points at *p* < 0.01; ^a−d^ different letters indicate significant differences among groups and time points at *p* < 0.001; ^a−e^ different letters indicate significant differences among groups and time points at *p* < 0.0001

**Fig. 5:**
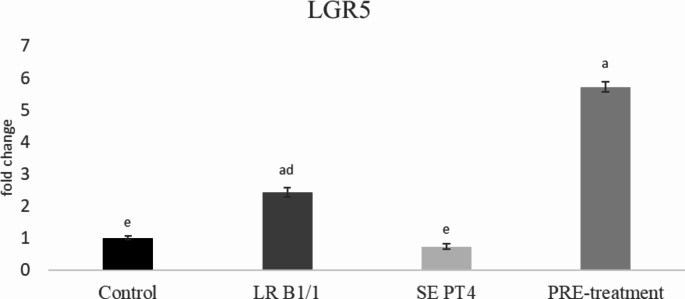
Relative gene expression for LGR5 receptor protein in the ileal explant. ^a−d^ different letters indicate significant differences among groups and time points at *p* < 0.001; ^a−e^ different letters indicate significant differences among groups and time points at *p* < 0.0001

**Fig. 6:**
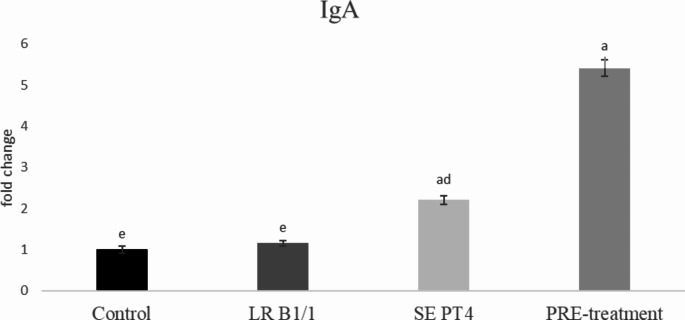
Relative gene expression for IgA in the ileal explant. ^a−d^ different letters indicate significant differences among groups and time points at *p* < 0.001; ^a−e^ different letters indicate significant differences among groups and time points at *p* < 0.0001

**Fig. 7:**
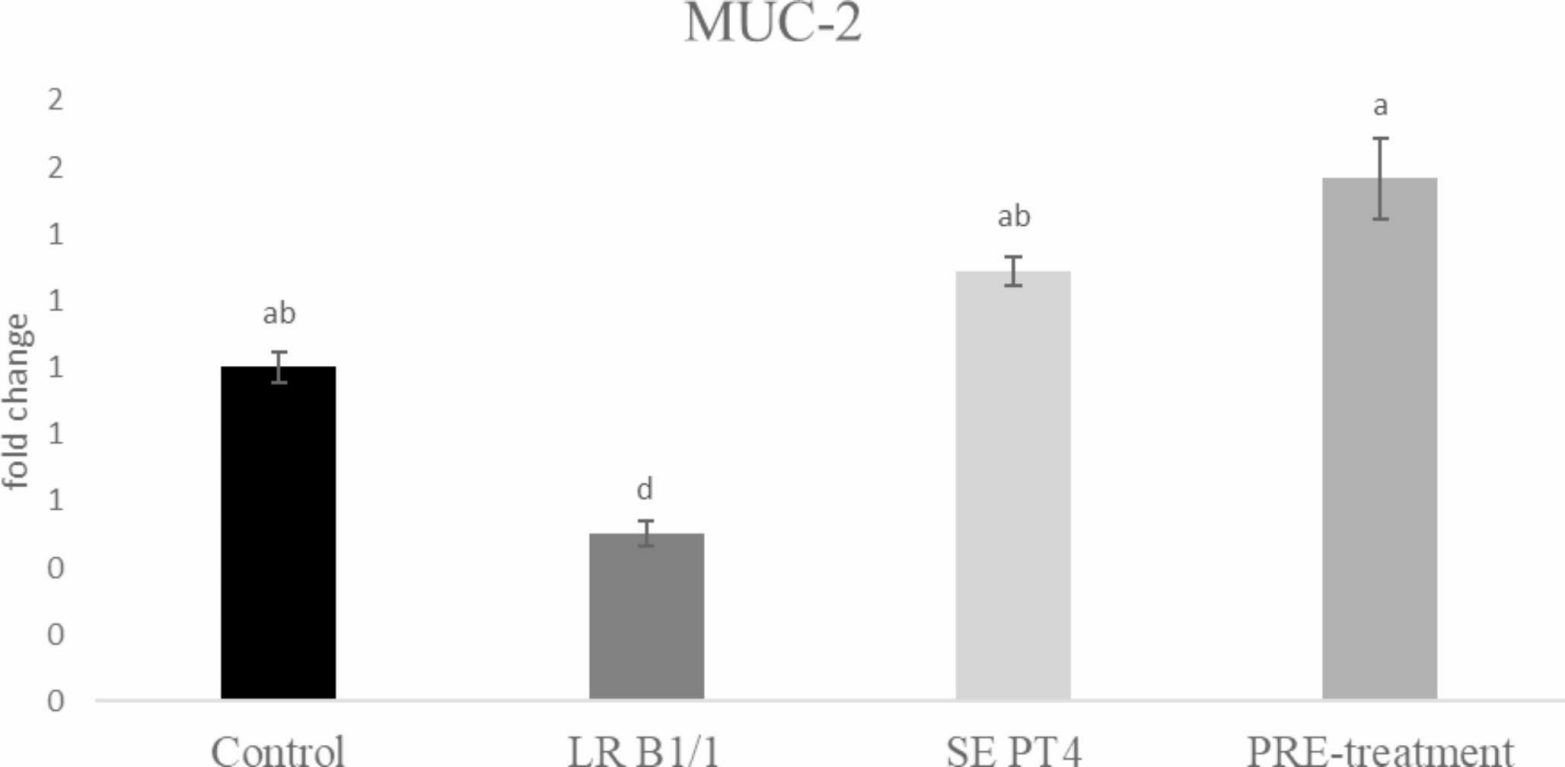
Relative gene expression for MUC-2 in ileal explant. ^a−b^ different letters indicate significant differences among groups and time points at *p* < 0.05;^a−d^ different letters indicate significant differences among groups and time points at *p* < 0.001

**Fig. 8:**
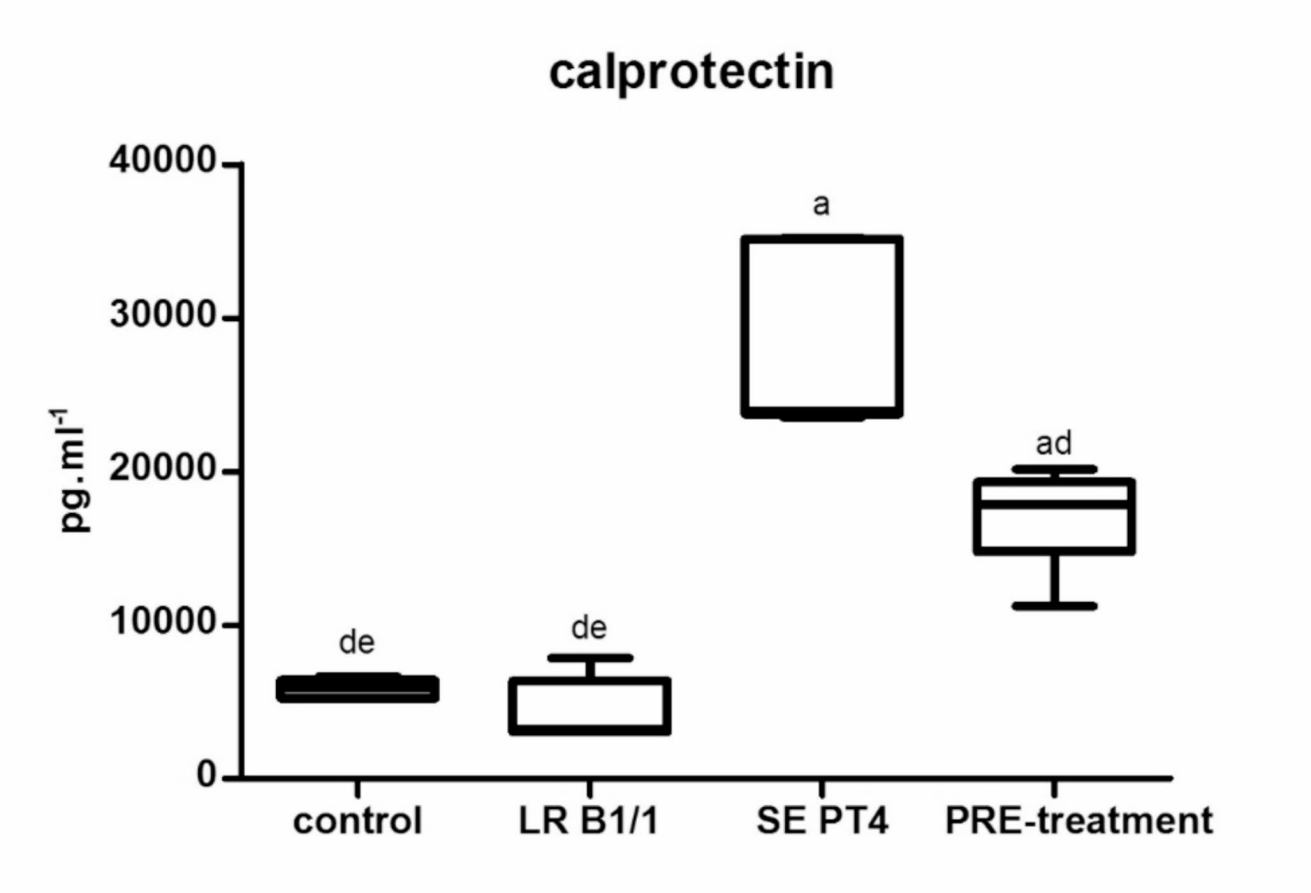
The distribution of the calprotectin concentrations (pg/ml) in ileal explant. The plots show the median (line within the box), 25th and 75th percentiles (box) and minimal and maximal values (whiskers). ^a−d^ different letters indicate significant differences among all groups and time points at *p* < 0.001; ^a−e^ different letters indicate significant differences among groups and time points at *p* < 0.0001

Relative gene expression of IL-15 was markedly up-regulated in PRE-treatment group compared to other groups (*p* < 0.0001) (Fig. 1). Similarly, relative gene expression for TGF-β4 was mainly up-regulated in the same group in comparison with other groups (*p* < 0.0001) (Fig. 2). The relative expression for E-cadherin was markedly up-regulated in LR B1/1 and control group compared to the SE PT4 (*p* < 0.0001) and PRE-treatment group (*p* < 0.01) (Fig. 3). The relative expression for tight junction protein - occludin was up-regulated markedly in both probiotic group in comparison with control as well as SE PT4 (*p* < 0.001; *p* < 0.0001) group (Fig. 4). On the other hand, relative expression for LGR5 gene was up-regulated in PRE-treatment group compared to other groups (*p* < 0.001; *p* < 0.0001) (Fig. 5). The same tendency was observed for IgA gene expression, which was significantly up-regulated mainly in PRE-treatment group in comparison with SE PT4 (*p* < 0.001) and other groups ((*p* < 0.0001) (Fig. 6). The level of gene expression for MUC-2 was relatively the same in all groups, except for the LR B1/1 group, where it was significantly down-regulated compared to the other groups (*p* < 0.0001) (Fig. 7). Significantly lowest calprotectin concentration was obtained in both non-infectious groups (control and LR B1/1), while the highest value was recorded in group SE PT4 compared to other groups (*p* < 0.001; *p* < 0.0001) (Fig. 8).

## Discussion

Salmonellosis is one of the most widespread poultry infections, which results in substantial economic losses and at the same time contaminated poultry products pose a threat to public health. Moreover, they can lead to zoonotic infections (Ijaz et al. 2021). In recent years, the use of alternative preventive strategies, including the use of probiotic bacteria, which can protect poultry against *salmonella* infections, has gained importance (Šefcová et al. 2023).

The testing of probiotic bacteria as well as their combinations has a long history (Ozen and Dinleyici 2015). Nevertheless, it is still true that one of the most important aspects of the probiotics used is the ability to positively modulate the animal’s immune system.

In the terms of previous statement, in our experiment we also observed the effect of the new probiotic isolate *L. reuteri* B1/1 in the pre-treatment on the ex vivo chicken ileal explant model on the specific parameters of local mucosal immune response after subsequent infection with *S*. Enteritidis PT4. We found that pre-treatment with *L. reuteri* B1/1 initiated an increased level of gene expression for both pro-inflammatory (IL-15) and anti-inflammatory (TGF-β4) cytokine. In general, pro-inflammatory IL-15 is a pleiotropic cytokine with a wide range of biological functions, including the initiation of inflammatory and protective immune responses to microbial agents by modulating immune cells of both the innate and adaptive immune systems (Perera et al. 2012). In the same way, binding of *salmonella* to the intestinal cells of mammals usually triggers the production of IL-15 as well as other pro-inflammatory cytokines (Fasina et al. 2008), but on the contrary, their production varies considerably depending on the breed of broiler chickens and is suppressed in some breeds (Kaiser et al. 2022). The gut is a TGF-β-rich environment where most cell types produce and respond to this cytokine. At the same time, in the intestinal environment, TGF-β plays a major role in the regulation of inflammation by coordinating balanced responses in the intestinal mucosa (Konkel and Chen 2011). It is well known, that there are two important signaling pathways in the regulation of intestinal immune responses: the Toll-like receptor/signal transducer and activator of transcription 3 (TLR/STAT3) pathway and the NF-κB p65 pathway. During inflammation, stimulation of TLR4 by lipopolysaccharide from pathogens further activates STAT3, which increases the production of pro-inflammatory cytokines that can stimulate the nuclear translocation of p65 and trigger the production of other pro-inflammatory cytokines such as IL-15 (Giridharan and Srinivasan, 2018). In this regard, authors Rastori and Singh (2022) described the observed mechanism of immunomodulatory action of limosilactobacilli in the intestine by means of their communication through membrane receptors TLR-6 and TLR-2, which are expressed on macrophages and dendritic cells, thereby stimulating the differentiation of individual T cell subsets. Moreover, the mentioned probiotic bacteria are able to influence the intracellular pathways of immune cells (e.g. macrophages) through MAP kinases, which either activate or suppress transcription factors, STAT, NF-kB, which can modulate the production of cytokines.

On the other hand, in our study expression of both cytokines were significantly down-regulated in infected group, which may reflects the effort of *Salmonella* Enteritidis not to provoke an inflammatory reaction. Conversely, Whitanage et al. (2005) observed increased expression of TGF-β4 in chickens infected with *S*. Typhimurium. This finding may be explained by the fact that there are differences in cytokine responses to different *salmonella* strains, which has been confirmed by several studies (Kogut et al. 2006; Swaggerty et al. 2006; Tang et al. 2018; Shaji et al. 2023). In addition, in the case of *salmonella*, the coordination of immune responses is strongly controlled by cytokines (Kaiser et al. 2022), so it is essential to modulate their production. Based on the above results, we may confirm the ability of the applied probiotic strain to modulate and potentiate the production of cytokines and thus contribute to the balance between pro- and anti-inflammatory mediators under ex vivo conditions. Similarly, in our previous study, the tested probiotic strain *L. fermentum* CCM 7158 demonstrated the potential to stimulate the expression of both pro-inflammatory and anti-inflammatory cytokines under different conditions in a porcine ex vivo model, on the other hand, second tested strain *L. reuteri* B1/1 manifested immunomodulatory potential, as it was able to suppress the pro-inflammatory response to *E. coli* lipopolysaccharide challenge (Kiššová et al. 2024). Also, Cao et al. (2013) reported, that the application of *Enterococcus faecium* stimulated an increase in the levels of anti-inflammatory IL-4 as well as pro-inflammatory IL-6 in the jejunal mucosa of broiler chickens. However, on the other hand, Zhen et al. (2018) found that *Bacillus coagulans* significantly reduced the levels of the pro-inflammatory cytokines TNF-α and IFN-γ in chickens infected with *Salmonella* Enteritidis.

These findings indicate that the detailed mechanism of action of each potential probiotic strain could be thoroughly investigated by using ex vivo models, which additionally allow to reduce the number of experimental animals in terms of the ARRIVE guidelines. Moreover, the results of several studies indicate that intestinal explants can faithfully mimic the specific production of cytokines in the intestine (Osaki and Mills 2016; Russo et al. 2016; Zhang et al. 2017), which was also confirmed in our experiment.

Basically, intestinal explants are cultures that are made from fragments of intestine or intestinal mucosa and maintained ex vivo to preserve the most important properties of organs in living animals, with the main advantage being a polarized and layered structure where physiological cell-cell interactions take place (Mátis et al. 2024a). In addition, they allow their safety to be initially tested before further in vivo testing. The chicken ileal explant model was successfully applied by Mátis et al. (2024a) in a recent study the possible immunmodulatory effect of cathelicidin-2 host defense peptide was examined. This may also indicate the wide possibilities of using animal intestinal explants in the prevention and treatment of gastrointestinal disorders while maintaining the specific conditions of culturing the explants (Stafford et al. 2016; Roselli et al. 2017; Van der Weken et al. 2021).

In the case of *salmonella* infection, targeting the innate immune system of young chickens is particularly important because their adaptive immune system is not fully developed and is not able to mount an effective immune response, and thus the host defense ultimately relies on innate immunity. In this terms, the composition of the intestinal microbiota can definitely contribute to the development of the innate immune response, as it also provides competition to invasive *Salmonella* serovars in the intestine (Meijerink 2021). Shibat El-hamd and Ahmed (2016) observed that the administration of a commercial probiotic preparation in dose 10^8^ CFU/mL (combination of several probiotic strains including *L. plantarum*,* L. acidophilus*,* Saccharomyces cerevisiae*) improved the immune response in chickens challenged with *Salmonella* Enteritidis.

The production of secretory IgA and gel-forming mucin-2 constitutes essential elements of mucosal immunity and contributes to the formation of a mucus barrier protecting against the invasion of pathogens (Grondin et al. 2020). In this context, several authors have observed that also the administration of probiotic bacteria can improve the balance of intestinal microorganisms, increase mucus secretion and reduce the degradation of TJ proteins caused by the presence of pathogenic bacterial strains (Ruemmele et al. 2009; Cristofori et al. 2021). In the case of the pre-treatment conditions, our tested lactobacilli strain *L. reuteri* B1/1 significantly stimulated gene expression for both mentioned parameters of mucosal immunity compared to the infected group, which may indicate an improvement in the ability of the mucous membrane to respond to bacterial pathogens.

It is widely known that maintaining a functional intestinal barrier is essential because it represents the first line of defense against infection by pathogenic microorganisms. In this context, TJ proteins such as occludin associated with epithelial cells ensure the integrity of the intestinal barrier by acting as a fence that prevents bacterial translocation (Chang et al. 2020). Cadherins are transmembrane proteins that mediate cell adhesion, with E-cadherin being a major component of adherens junctions. Down-regulation of E-cadherin expression in the intestine is associated with disruption of intestinal barrier function and homeostasis, indicating development of the pathogenesis of gastrointestinal diseases (Ghosh et al. 2021). Subsequently, LGR5 as a biomarker of active stem cells, which, in addition to other functions, ensures the restoration of the intestinal epithelium (Hou et al. 2020). Similarly, Zhang et al. (2014) reported that *salmonella* infection induced disruption of epithelial tight junctions in an infected murine intestinal organoid. In accordance with the previous study, down-regulation of E-cadherin and LGR5 gene expression was observed in the infected group.

On the other hand, our probiotic strain positively modulated gene expression for TJ protein– occludin, transmembrane junction protein E-cadherin as well as LGR5 as a reaction to the presence of *salmonella* in pre-treatment group. Based on these findings, we hypothesize that application of *L. reuteri* B1/1 may enhance the function of intestinal mucosal epithelial cells during *salmonella* infection. Consistent with our findings, a study by Chang et al. (2020) confirmed, that supplementation of multi-strain *lactobacillus* probiotics increased the mRNA gene expression of occludin, zonula occludens-1, and mucin-2 in the ileum of broiler after *S. enterica* infection.

In human medicine, the determination of calprotectin protein is currently a new non-invasive method for evaluating the inflammatory process in the intestine. It is used in the diagnosis of diseases of the large intestine, cancer, as well as for the evaluation of the effectiveness of treatment (Jukic et al. 2021). Generally, its production increases during ongoing inflammation in the intestine, which was also confirmed in our experiment, where the production was the highest in the *S*. Enteritidis infected group. Similarly, other studies reported up-regulation of gene expression during infection with *Clostridium perfringens* or *Eimeria maxima* in the gut of broilers (He et al. 2019). On the other hand, *L. reuteri* B1/1 significantly reduced calprotectin production in ileal explants in the both lactobacilli group, which may indicate the ability of the probiotic strain to modulate its production according to current physiological or pathophysiological conditions. It is also an important fact, because in the case of the release of a large amount of calprotectin, which is accompanied by an overproduction of reactive oxygen species (ROS) and the subsequent secretion of pro-inflammatory cytokines, it can also result in a prolonged inflammatory reaction (Bourgonje et al. 2024). Therefore we hypothesize that its production may be controlled or modulated by probiotic bacteria.

In summary, we can conclude that the newly isolated probiotic strain *L. reuteri* B1/1 confirmed its ability to effectively modulate the mucosal immune response in pre-treatment conditions for *S*. Enteritidis PT4 infection in the chicken ileum explant model, which may bring wide possibilities for its use as a new probiotic strain in poultry farming. Moreover, our obtained results point to the fact that the new chicken ileum explant model could be a suitable model for investigating or testing the influence of natural substances such as probiotic bacteria in the interaction with the intestine as well as pathogenic microorganisms. The results of our study can contribute to a deeper understanding of the action of newly isolated probiotic bacteria at the intestinal level by using ex vivo models such as the chicken ileal explant, which are able to mimic in vivo conditions sufficiently. In addition, their use allows to reduce the number of animals used in an experiment.

## Data Availability

No datasets were generated or analysed during the current study.
